# Bilateral cortical representation of orgasmic ecstasy localized by depth electrodes^☆^

**DOI:** 10.1016/j.ebcr.2013.03.002

**Published:** 2013-04-13

**Authors:** Werner Surbeck, Alain Bouthillier, Dang Khoa Nguyen

**Affiliations:** a Section of Neurosurgery, Kantonsspital St. Gallen, Switzerland; b Section of Neurosurgery, Notre-Dame Hospital, Montreal, Canada; c Section of Neurology, Notre-Dame Hospital, Montreal, Canada

**Keywords:** Orgasm, Ecstasy, Cortical representation, Depth electrodes

## Abstract

While sexual arousal had been evoked during direct electrical stimulation (DES) of the right mesial temporal lobe and basal forebrain, isolated orgasmic ecstasy (OE) evoked by DES is not reported in the literature. We present the first case of isolated bihemispheric reproduction of OE by stimulation via depth electrode in a patient implanted for epilepsy.

## 1 Introduction

As sexual sensations are subjective and intimate, they are difficult to evaluate in laboratory conditions. Traditional studies on the brain basis of human sexual arousal and orgasm are mainly based on neurological patients presenting epileptic seizures with sexual manifestations or patients presenting sexual symptoms as a consequence of focal or disseminated lesions [1]. Introduction and advances in functional neuroimaging techniques, such as single photon emission tomography, positron imaging (PET), functional magnetic resonance imaging, and magneto-encephalography, have opened a new window for the study of sexual sensations both in healthy subjects and in subjects with sexual disorders [2]. However, direct electrical stimulation (DES) of cortical regions remains the gold standard for localization of brain functions [3]. While sexual arousal, in one case resulting in an orgasm, had been evoked during depth electrode stimulation mapping of the right mesial temporal lobe [4], [5], and varying degrees of sexual arousal were consistently elicited by electrical stimulation of basal forebrain structures [6], isolated orgasmic ecstasy (OE) as the first and only symptom of local cortical stimulation is not reported in the literature. We report a case of isolated reproduction of OE by stimulation via depth electrode in a patient implanted for epilepsy.

## 2 Case report

**Fig. 1:**
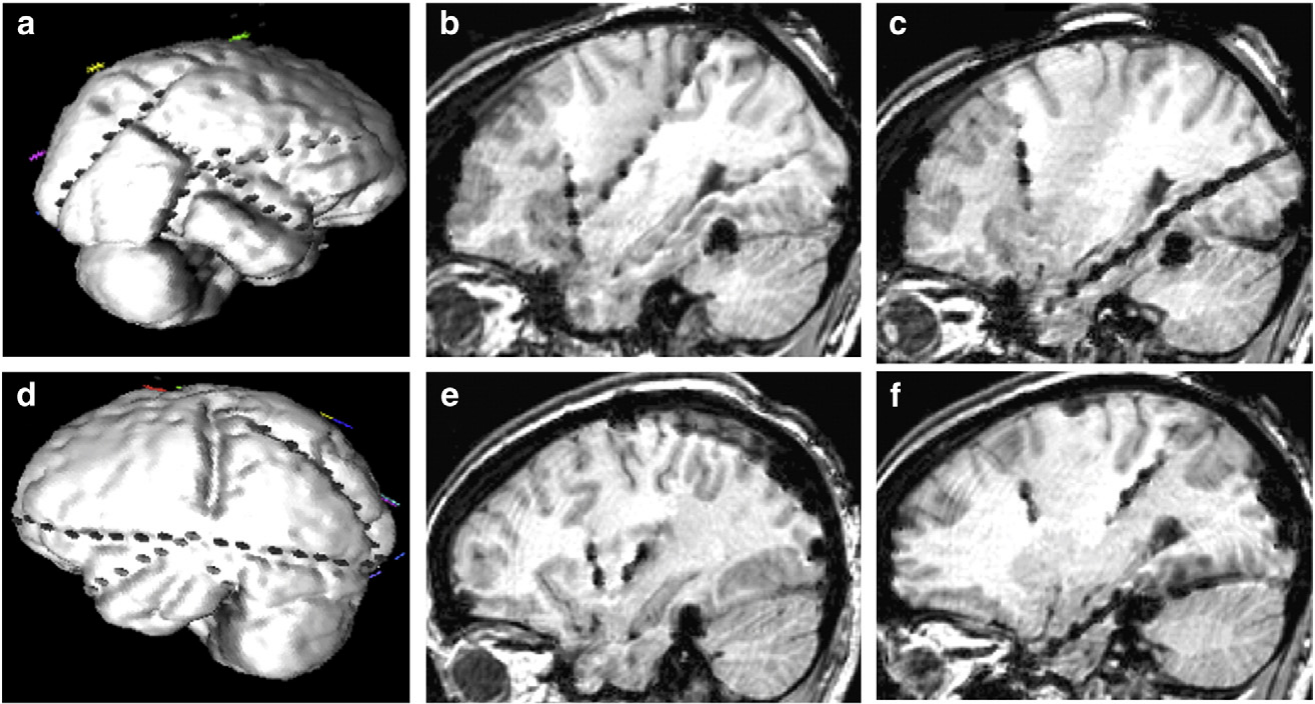
(a) Three-dimensional visualization of the right hemispheric subdural electrode arrangement. (b) Sagittal MRI view of the anterior and posterior insular electrodes on the right side. (c) Sagittal MRI view of the hippocampal electrode on the right side. (d) Three-dimensional visualization of the left hemispheric subdural electrode arrangement. (e) Sagittal MRI view of the anterior and posterior insular electrodes on the left side. (f) Sagittal MRI view of the hippocampal electrode on the left side.

The patient was a 49-year-old, right-handed woman with no significant past medical history except for medically intractable epilepsy since age 15 years. Seizures were characterized by altered consciousness and oral automatisms followed by postictal confusion. While some seizures were not preceded by any aura, some may start with visual symptoms (flashing lights), déjà vu, or an orgasmic feeling. Secondarily tonic-clonic seizures were rare. Magnetic resonance imaging showed a very discrete atrophy of the left hippocampus. Video-scalp electroencephalography (EEG) monitoring disclosed bilateral temporal spikes. Several seizures were recorded with diffuse EEG changes and maximum build-up more frequently over the left than the right temporal region. FDG PET was normal. Suspecting a possible posterior focus with propagation to the temporal lobes, an invasive electrode study was subsequently performed. A total of 6 depth electrodes, 15 subdural strip electrodes and a small subdural grid electrode were inserted to sample the medial, lateral, inferior, and posterior temporal regions and the insular, occipital, parietal, and inferior frontal regions bilaterally (Fig. 1).

**Fig. 2:**
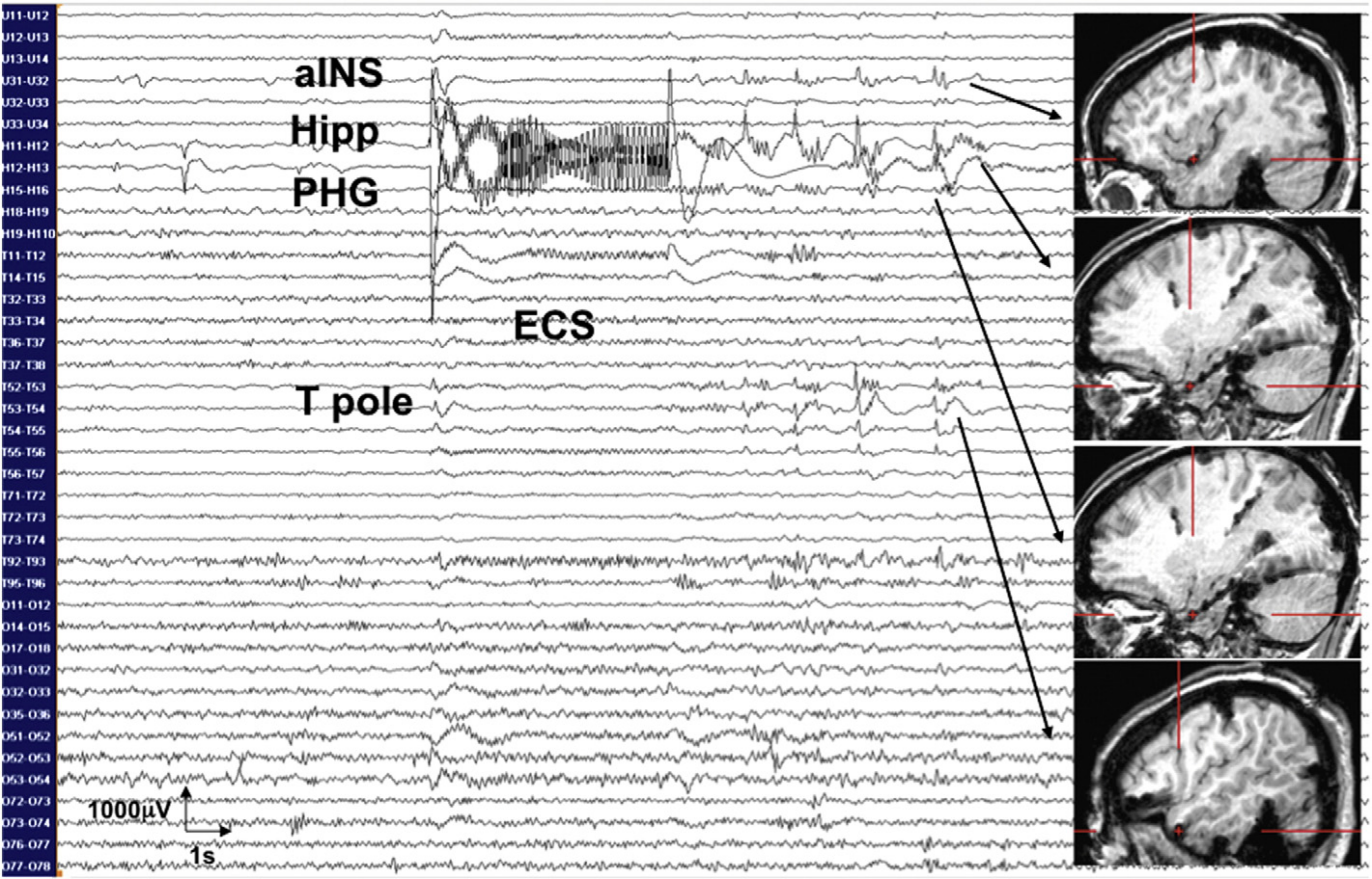
The patient reported orgasmic ecstasy following the stimulation of the left hippocampus (Hipp) at 3 mA. This stimulation was followed by an 18-second afterdischarge over the left hippocampus (Hipp), the parahippocampal gyrus (PHG), the temporal pole (T pole), and the anterior-inferior insula (aINS).

**Fig. 3:**
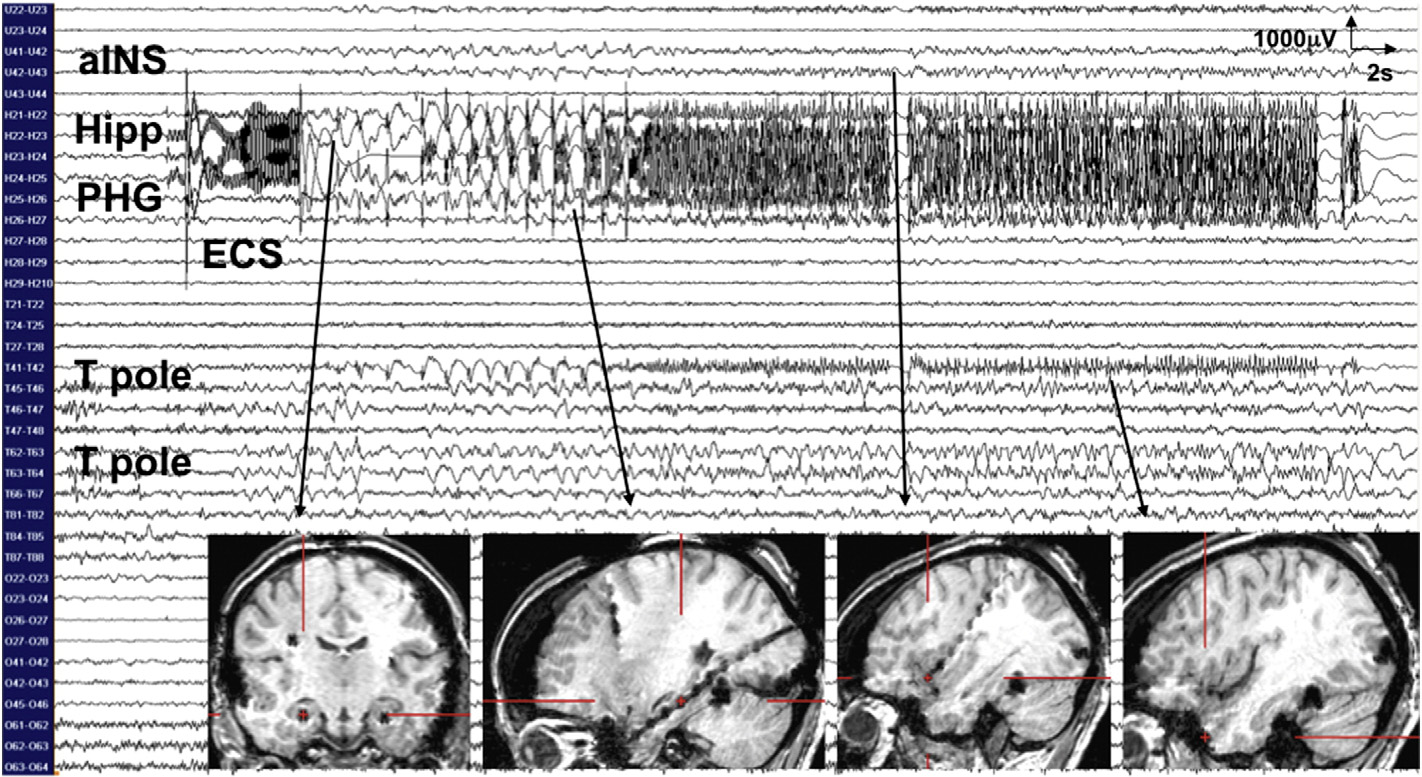
Stimulation of the right hippocampus at 1 mA generated orgasmic ecstasy while triggering a 45-second seizure discharge over the right hippocampus (Hipp), the parahippocampal gyrus (PHG), the temporal pole (T pole), and the anterior insula (aINS).

As part of the intracranial EEG monitoring study, electrical stimulation was performed for cortical mapping and for the identification of contacts which could reproduce the patient's auras. The parameters used were as follows: stimulus frequency of 50 Hz with a pulse width of 100 ms, a stimulus intensity of 1–10 mA, and a stimulus train duration of 5 s (Grass S88 Stimulator, Grass Instruments, Natick, MA). Stimuli were administered with an intervening rest interval of at least 1 min, and afterdischarges were assessed at each stimulation. The patient reported an OE following the stimulation of the left hippocampus at 3 mA. This stimulation was followed by an 18-second afterdischarge over the left hippocampus, the parahippocampal gyrus, and the anterior-inferior insula (Fig. 2). The identical sensation was reproduced when stimulation was repeated a second time at 3 mA with a similar 17-second afterdischarge. Stimulation of the right hippocampus at 1 mA generated the same orgasmic sensation while triggering a 45-second seizure discharge over the right hippocampus, parahippocampal gyrus, temporal pole, and anterior insula (Fig. 3). In the end, no resection was performed as spontaneous seizures were found to originate from both right and left hippocampi as well as the left temporal neocortex and the left cuneus.

## 3 Discussion

Because of its intimate nature, the representation of sexual functions in the cerebral cortex has received little attention until the second half of the 20th century. Penfield and Rasmussen, who greatly enhanced the understanding of cortical localization by extensive and consequent use of DES during surgery, succeeded only in two patients to demonstrate sensory genital hallucinations from stimulation of the postcentral gyrus within the interhemispheric fissure [7]. They had never been able to evoke erotic sensations of any type. Such sensations, although rare, have been known for a long time in patients with epilepsy [8]. Ictal symptoms may range from erotic feelings to sexual arousal and even OE. All of them are referred to as sexual auras (SAs) [9]. It seems reasonable to assume that SAs are caused by epileptic activation of the same brain regions that produce the physiological correlates. According to the existing case reports with an identified epileptogenic focus, SAs are strongly associated with temporal lobe epilepsy (TLE) [10]. Sexual auras are exceptionally reported in patients with parietal lobe epilepsy. In these cases, activation of the primary sensory center of the genitalia leading to orgasm is assumed [11]. In patients with TLE and SAs, there is significant lateralization predominance of the epileptogenic region to the right hemisphere [10]. Furthermore, three out of four reported patients with SAs are female, suggesting that the neural organization of psychosexual behavior differs in human male and female brains [5], [10]. This may be partly explained by the fact that orgasm is less tightly linked to perceived physiological cues in women than in men, but rather on a mental basis [12]. In contrast with the rare phenomena of sexual manifestations during temporal lobe seizures, alterations in interictal sexual behavior have been frequently reported in patients with epilepsy, particularly TLE. The most common interictal sexual dysfunction associated with TLE is hyposexuality [13]. This underscores the prominent role of the temporal lobes in human sexual function. Consistent with the above mentioned right hemispheric dominance of TLE with SAs, the few reports of sexual arousal and OE evoked by DES of the temporal lobe have been reported only from right-sided stimulations. During depth electrode stimulation mapping in two patients with complex partial seizures and SAs, right temporomesial stimulation during intraoperative electrocorticography triggered their habitual auras of sexual arousal [5]. In another patient implanted with invasive intracranial electrodes for complex partial seizures, habitual seizures with complex motor automatism followed by erotic feelings and OE were reproduced by stimulation of the right amygdala with the induction of seizures involving the ipsilateral amygdala, hippocampus, and parahippocampal gyrus [4]. Finally, Gloor [14] reported sexual arousal in a female patient with epilepsy during electrical stimulation of the right amygdala. The stimulation was followed by afterdischarges involving the amygdala and other limbic structures of the right temporal lobe. Limitations of these observations include limited electrode sampling and therefore restricted or no monitoring of stimulation-induced afterdischarges.

Our case report provides further insight into the generation of OE. For one, such a response can be evoked bilaterally. Second, activation of a larger network appears to be necessary in order to generate such sensations. On the left side, OE was only reported by the patient when stimulation of the hippocampus was accompanied by afterdischarges over the hippocampus, the parahippocampal gyrus, and the anterior-inferior insula of the same side. Stimulation of the right hippocampus generated the same orgasmic sensation by triggering a seizure discharge over the ipsilateral right hippocampus, parahippocampal gyrus, temporal pole, and anterior insula. These observations indicate bilateral representation of OE within the region of the hippocampus, parahippocampal gyrus, temporal pole, and anterior insula. Studies of ecstatic epileptic seizures have recently led to the proposal that ecstatic states are based on a hyperactivation of the anterior insula (although no electrode was in direct contact with the insula for any of these patients) [15], [16], [17]. Ecstatic epileptic seizures share many features with OE, such as intense positive feelings and subjective slowing of time. Our observation supports the participation of the anterior insular cortex in ecstatic states. The anterior insula could be a nodal point of diverse functional networks encoding ecstatic states. It remains unclear, however, why stimulation of the anterior insula, for this patient or in previous insular cortical stimulation studies, has failed to evoke OE [18], [19], [20], [21], [22]. Possible explanations include downsampling of the anterior-inferior portion of the insula during SEEG explorations due to the abundance of vascularization at that level limiting the implantation of orthogonal electrodes, failure to increase stimulus intensity to activate a larger network when obtaining visceromotor- or viscerosensory-evoked responses at lower intensity, or that the hippocampus is the main node of the network which must be activated maximally to generate OE.

One limitation of our case report is the lack of sampling of the basal forebrain, an area not usually sampled in studies of epilepsy for clinical purposes. In 1964, Heath reported varying degrees of sexual arousal in 54 patients elicited by electrical stimulation of the “septal region” [6]. However, sexual orgasm was never produced. Heath referred to the septal region in men as an anatomical site close to the midline and extending from the rostral tip of the frontal horn of the lateral ventricle to the level of the anterior commissure. This area includes several basal forebrain structures including the subcallosal cortex, medial parts of the ventral striatum, the diagonal band of Broca, parts of the medial preoptic area, and the septal nuclei [23]. Based on animal studies, the latter is known as a pleasure center. Rats, for example, would work to stimulate brain electrodes implanted in the septal nuclei [24]. There is some disagreement about whether these stimulations elicited pleasure or only craving to obtain more stimulation [25]. Even though the septal nuclei may be involved in sexual arousal, they seem to be insubstantial for OE.

## 4 Conclusion

Observation from this case study and previously reported cases suggests that OE involves the unilateral activation of a network comprising the amygdala, hippocampus, the parahippocampal gyrus, the temporal pole, and the anterior inferior insula.
